# A note on the larva of *Chalcophora japonica chinensis* (Coleoptera, Buprestidae) based on morphological characters and molecular data

**DOI:** 10.3897/zookeys.944.37765

**Published:** 2020-06-30

**Authors:** Zhonghua Wei, Liumei Zhang, Aimin Shi

**Affiliations:** 1 The Key Laboratory of Southwest China Wildlife Resources Conservation of the Ministry of Education, College of Life Sciences, China West Normal University, 637009, Nanchong, Sichuan Province, China The Key Laboratory of Southwest China Wildlife Resources Conservation of the Ministry of Education, College of Life Sciences, China West Normal University Nanchong China; 2 College of Life Sciences, Hebei University, 071002, Baoding, Hebei Province, China College of Life Sciences, Hebei University Baoding China

**Keywords:** *
Chalcophora
*, China, DNA marker, jewel beetles, larval morphology

## Abstract

Larvae of *Chalcophora japonica chinensis* Schaufuss, 1879 were collected from within dead trunks in Hubei Province, China, in February 2019. These specimens created an opportunity to provide the first description of the larval stage of this subspecies; The larva is described and illustrated based on morphological characters and DNA barcoding.

## Introduction

To date, nine species and 24 subspecies of the genus *Chalcophora* Dejean, 1833 have been distributed from Palaearctic region. Of these, two species and seven subspecies have been recorded from China (Kubáň 2016). The larva of *Chalcophora virginiensis* (Drury, 1770), a North American species (Maier and Ivie 2013), was simply illustrated by Böving and Craighead (1931), who provided only three simple drawings, without a detailed description. Subsequently, the larvae of three *Chalcophora* species were described from the Palaearctic region and the interspecific differences of larval morphology were summarized by Bílý (1984). The taxonomic characters of larvae of known chalcophine genera were summarized by Bílý and Volkovitsh (2003).

Larvae of *Chalcophora* differ from known chalcophorine larvae by the following characters, as summarized by Bílý and Volkovitsh (2002, 2003): (1) anterior margin of labium with narrow microsetal area; (2) isolated sclerite of maxillary cardo with two short setae and five campaniform sensilla; (3) anterior sclerotized area of pronotal groove umbrella-shaped, transverse, with broadly rounded anterior margin.

Recently, DNA barcodes and markers have been used as an alternative approach for identification of arthropod stages, such as the identification of immature terrestrial (Caterino and Tishechkin 2006; Ekrem et al. 2007; Andrić et al. 2014) and aquatic arthropods (Miller et al. 2005). The complete mitochondrial genome of *Chrysochroa fulgidissima* has been determined by Hong et al. (2009).

Herein, we provide a morphological description and DNA barcoding of the larva of *Chalcophora japonica chinensis* Schaufuss, 1879.

## Materials and methods

The specimens were examined using a Nikon SMZ800 stereomicroscope. The habitus images were taken using a Canon EOS 5D camera combined with a Canon MP-E65 mm macro lens.

The total genomic DNA was extracted from larval and adult tissues using EZNA Insect DNA Kit (Omega Bio-tek, USA). One fragment of the mitochondrial protein-coding gene (COI) was amplified from both larva and adult. The DNA barcode region (Hebert et al. 2003) was amplified using primers LCO1490 and HCO2198 (Folmer et al. 1994). The dataset used contained *Chrysochroa fulgidissima* (GenBank 7944365) as the outgroup. Sequences were aligned using the ClustalW algorithm (Thompson et al. 1994), as implemented in BioEdit v. 7.0.9.0. (Hall 1999). The maximum likelihood tree was constructed using the GTR+R model in Mega v. 6.06. The examined specimens are deposited in the China West Normal University (**CWNU**). Three adult specimens of *Chalcophora yunnana yunnana* were collected from Yingjiang of Yunnan province. The morphological terminology follows that used in the papers of Volkovitsh and Bílý (1997, 2001) and Bílý and Volkovitsh (2002, 2003).

## Taxonomy

### Key to known larvae of *Chalcophora* from Asia

**Table:** 

1	Pronotal and prosternal asperate area consisting of transverse, short asperities and wrinkles	**2**
–	Pronotal and prosternal asperate area consisting of transverse, long or elongate asperities and lines	**3**
2	Y-shaped pronotal grooves with shorter branches, angle between branches about 30°, branches 1.3 times as long as common part; anterior margin of labrum slightly emarginate (Fig. 3C)	***C. intermedia***
–	Y-shaped pronotal grooves with longer branches, angle between branches about 30°, branches 2.5 times as long as common part; anterior margin of labrum slightly arcuate (Fig. 3B)	***C. mariana***
3	Y-shaped pronotal grooves slightly bent at middle, angle between branches about 25°, branches 2.3 times as long as common part (Fig. 3A)	***C. detrita***
–	Y-shaped pronotal grooves straight, angle between branches about 23°, branches 2.5 times as long as common part (Fig. 3D)	***C* . *japonicachinensis***

#### Chalcophora japonica chinensis

Taxon classificationAnimaliaColeopteraBuprestidae

Schaufuss, 1879

79B449B0-F331-5DB7-B265-69D0B41C0A97

**Figure 1.**
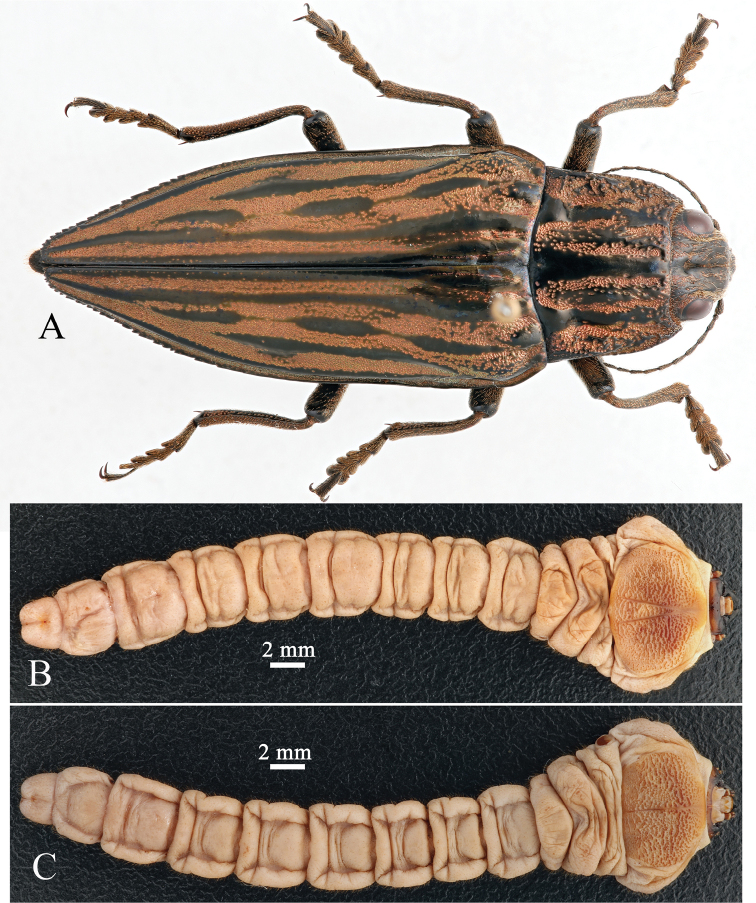
Habitus of *Chalcophora japonica chinensis* (Schaufuss, 1879) **A** adult, in dorsal view **B** larva, in dorsal view **C** larva in ventral view.

**Figure 2.**
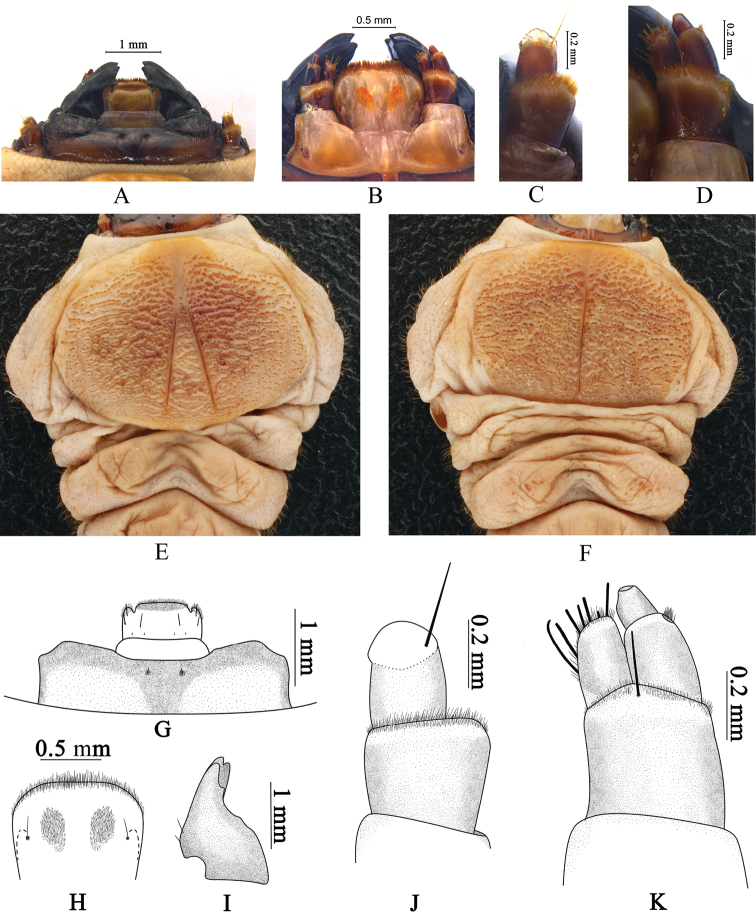
Larval morphology of *Chalcophora japonica chinensis* Schaufuss, 1879 **A, G** epistome and mouth parts, in dorsal view **B** mouth parts, in ventral view **C, J** antenna **D, K** maxilla, in ventral view **E, F** thorax, in dorsal and ventral views **H** prementum **I** left mandible.

**Figures 3.**
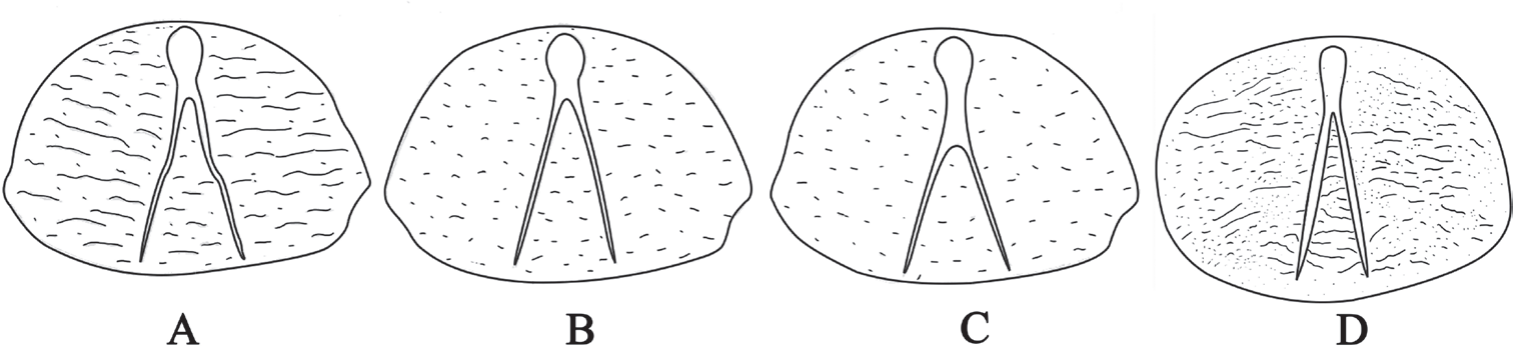
Larval pronotal plate of *Chalcophora* species (**A–C** from Bílý 1984) **A***C. detrita***B***C. mariana***C***C. intermedia***D***C. japonica chinensis*.

Figures 1B, C , 2A–K , 3D

Chalcophora chinensis Schaufuss, 1879: 480; Fairmaire 1888: 25; Kurosawa 1974: 174.
Buprestis sinica Jakobson, 1913: 780.

##### Distribution.

China (Anhui, Fujian, Guangdong, Guangxi, Henan, Hong Kong, Hubei, Hunan, Jiangsu, Jiangxi, Sichuan, Zhejiang provinces).

##### Larval description.

The description is based on what is probably two later-instar larvae. Body length 42.0–52.1 mm; width of prothorax 10.3–11.6 mm. Larval body shape of buprestid type. Body elongate (Fig. 1B, C); prothorax widest, distinctly wider than meso- and metathorax; abdomen nearly parallel-sided. Mouthparts brown; mandibles black; dorsal and ventral prothoracic plates yellow-brown; abdomen light brown (white when alive). Body with sparse, short, brownish setae. Thoracic legs absent.

***Head*.** Head prognathous (Fig. 2A, G), with mouthparts directed anteriorly, head capsule deeply retracted into prothorax. Stemmata absent.

***Labrum*** (Fig. 2A, G) distinctly transverse, 2.1 times as wide as long; anterior margin and isolated patches on lateral lobes bearing dense microsetae, middle parts slightly arched, anterolateral lobes well developed; lateral sides slightly arched; posterior margin (covered by nearly pellucid membranous anteclypeus) slightly narrower than anterior margin; dorsal surface glabrous; medial group of sensilla consists of one apical seta and two lateral campaniform sensilla; apical seta not reaching anterior margin; anterolateral sensilla consists of two trichoid sensillae.

***Anteclypeus*** (Fig. 2A, G) trapezoid, short, strongly transverse, 5.6 times as wide as long, membranous, glabrous; anterior margin nearly straight, weakly emarginate at middle; lateral sides distinctly arched.

***Epistome*** (Fig. 2A, G) strongly transverse, approximately 3.3 as wide as long; anterior parts dark brown, well sclerotized, posterior part brown, moderately sclerotized; lateral parts of anterior margin distinctly protruded anteriorly, middle parts distinctly emarginate; lateral sides bent; surface with two groups of short trichosensillae situated in shallow depressions in middle parts.

***Antennae*** (Fig. 2C, J) two-segmented, situated in deep incision, first antennomere approximately 1.8 times as long as second, approximately 1.2 times as long as wide, barrel-shaped, glabrous except anterior margin with dense microsetae; second antennomere distinctly shorter and narrower, nearly as long as wide, distinctly expanded anteriorly, anterior parts transparent membranous, glabrous; apical cavity deep, bearing a long erect trichosensillum, slightly longer than second antennomere.

***Mandibles*** (Fig. 2A, I) triangular, strongly sclerotized, black, approximately 1.1 times as long as wide; middle of incisor edge with an additional tooth; outer margin with one short erected seta above condyle.

***Maxillae*** (Fig. 2B, D, K). Cardo transverse, subquadrate, widest at base, approximately 2.8 times as wide as long, glabrous; latero-basal sclerite of cardo sclerotized, with five campaniform sensillae bearing two short, erect, brown setae. Stipes subquadrate, distinctly wider than long. First palpomere of palpus maxillaris approximately 1.8 times as long as second palpomere, barrel-shaped, distinctly longitudinal, glabrous, outer parts of apical margin with a few microsetae; second palpomere distinctly narrower, glabrous, subcylindrical, approximately 1.4 times as long as wide. Mala elongate, approximately 1.8 times as long as wide, external sensilla of apex composed of one campaniform sensillum, one short, peg-like seta, five long, robust setae, and a few microsetae; internally with three short, robust setae and a few microsetae along inner side. Prementum (Fig. 2H) trapezoidal, anterior margin arcuate and wider than base, with surface bearing two microsetal zones in middle parts and two long setae near sides of middle parts.

***Thorax*** (Figs 2E, F, 3D). Prothorax strongly expanded, widest at middle, approximately 1.7 times as long as total length of meso- and metathorax, approximately 1.7 times as wide as long; dorsal and ventral plates moderately sclerotized, strongly flattened, with transversely prolonged asperities and short winkles; dorsal plate with inverted Y-shaped groove with long, straight branches; angle between branches about 23°; branches 2.5 times as long as common part; anterior sclerotized part nearly umbrella-shaped, with rounded anterior margin; ventral plate with uniramous groove; lateral parts bearing short, brownish setae. Mesothorax nearly an inverted trapezoid, widest at anterior margin, approximately 4.5 times as wide as long; surface bearing short, brownish setae; spiracles cribriform, reniform, strongly transverse, much larger than those of abdomen, approximate 2.2 times as wide as long, situated on sides of anterior parts; surface with dense, longitudinal, straight lines. Metathorax nearly an inverted trapezoid, widest at middle, approximately 2.2 times as wide as long; middle part of anterior margin distinctly protruded anteriorly; posterior margins distinctly emarginate; surface bearing sparse, short, brownish setae.

***Abdomen*.** All segments transverse, slightly wider than long; surfaces of all segments with sparse, short, brownish setae; all dorsal parts of segments distinctly convex; dorsal parts of segments II–VIII each with distinctly transverse, deep groove on posterior parts; dorsal parts of segment IX with transverse, shallow groove before the base. Segment I nearly subtriangular, widest behind middle, approximately 2.3 times as wide as long; anterior margin distinctly protruded anteriorly; surface of dorsal parts with a few longitudinal wrinkles. Segments II–IX subquadrate, approximately 1.2–2.3 times as wide as long; segment X well developed and posteriorly oriented, with paired, oval lobes divided by longitudinal, shallow anal cleft. Segments I–VIII with large spiracles, gradually becoming smaller posteriorly, spiracles of segment VIII smallest; segments IX and X without spiracles.

##### Specimens examined.

***Larvae***: 2 ex., China, Hubei, Xiangyang, Baokang, Chengdong, Mingjiachang, 15-II-2019, Jie Deng leg., CWNU, taken from the stems of dead tree. ***Adults***: 5♂ 5♀, China, Guanngxi, Guilin, Ziyuanzhongfeng, 26-VII-2018, Yusong Huang leg., CWNU; 1♂ 1♀, Guangdong, Wengyuan, Nanpu, 5-X-2019, Hua Zhang leg., CWNU; 1♂, China, Jiangsu, Nangjiang, Zhongshanling, 18-IV-2019, Zhixin Chou, CWNU.

## Results and discussion

**Figures 4.**
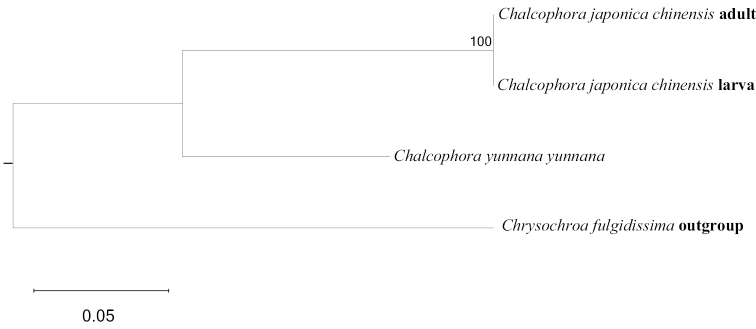
Maximum Likelihood tree of *Chalcophora* from China based on COI.

There are two species and seven subspecies of *Chalcophora* recorded from China, accounting for 29% of the species known in the Palaearctic Region. Recently, some buprestids, including chalcophorine larvae, were described by Bílý and Volkovitsh (2002, 2003, 2005), Volkovitsh and Bílý (2001, 2015), and Bílý et al. (2013). In this study, data supporting the utility of COI sequences analysis is provided for identifying species at developmental larval stages. The larval COI sequence clustered together with the adult *C. japonica chinensis* sequences with high bootstrap support (Fig. 4), enabling us to identify the larvae as *C. japonica chinensis*.

## Supplementary Material

XML Treatment for Chalcophora japonica chinensis

## Supplementary materials

Supplementary material 1 Detailed results of COI sequences dataset Data type molecular data https://binary.pensoft.net/file/427157This dataset is made available under the Open Database License (http://opendatacommons.org/licenses/odbl/1.0/). The Open Database License (ODbL) is a license agreement intended to allow users to freely share, modify, and use this Dataset while maintaining this same freedom for others, provided that the original source and author(s) are credited.Zhonghua Wei, Liumei Zhang, Aimin Shi
